# A Systematic Review of Factors Associated With Treatment Engagement and Outcome for Women in the Perinatal Period Receiving Individual Cognitive Behavioral Therapy (CBT) for Depression, Anxiety, and Trauma-Related Disorders

**DOI:** 10.1155/da/3698331

**Published:** 2025-09-18

**Authors:** Natalie A. Simon, Jenna Evans, Darcy Bispham, Ffion Williams, Jonathan Jones, Neil P. Roberts, Cerith S. Waters

**Affiliations:** ^1^South Wales Clinical Psychology Training Program, School of Psychology, Cardiff University, Cardiff, Wales, UK; ^2^Perinatal Community Mental Health Service, Cardiff and Vale UHB, Cardiff, Wales, UK; ^3^Division of Psychological Medicine and Clinical Neuroscience, Cardiff University, Cardiff, Wales, UK

## Abstract

Depression, anxiety, and trauma-related disorders commonly occur in the perinatal period, with high rates of comorbidity, and potentially adverse outcomes for women and children. Cognitive behavioral therapy (CBT) is an effective treatment, however less than half of new mothers experiencing symptoms seek treatment. This review was focused on factors affecting treatment engagement and outcome in a clinical perinatal population. A mixed-methods systematic review was conducted according to Cochrane Collaboration Guidelines. We included randomized controlled trials (RCTs) of individual CBT where at least 70% of women met diagnostic criteria for depression, anxiety, or trauma-related disorders during the perinatal period. Information on, and factors associated with treatment engagement, satisfaction, therapeutic alliance were examined as risk ratios (RRs). Twenty-eight studies relating to 19 RCTs, with 2557 participants were reviewed. Most studies examined CBT adapted to the perinatal context. Engagement was good overall, and high levels of satisfaction and therapeutic alliance were reported. A relationship was demonstrated between engagement and outcome in three studies. Dropout did not differ for studies of CBT compared to treatment as usual (TAU), but there was greater dropout for CBT across four studies where the guiding therapist/coach had a minimal level of psychological therapy training and qualifications (*k* = 4; *n* = 675; RR 2.38; CI 1.17–4.83). Findings indicate the importance of engagement, which may be optimized by adapting CBT to be relevant to the unique challenges faced by women in the perinatal period, and through therapeutic provision from therapists with at least a moderate amount of psychological therapy training and qualifications.

## 1. Introduction

The perinatal period typically spans from the beginning of pregnancy to up to 1 year after birth. Prevalence rates for perinatal mental health conditions exist, though due to the variety of methodological approaches used in the literature their assimilation is challenging [1]. Perinatal depression prevalence has been estimated at 11.9% in one systematic review [2], and as high as 26.3% in another [3], and a clinical diagnosis of any anxiety disorder during pregnancy as 15.2%, and postnatally (1–24 weeks) as 9.9%, with higher rates reported for low- to middle-income countries [4]. Tokophobia, or fear of childbirth (FOC), is estimated to affect around 14% of pregnant woman [5], and post-traumatic stress disorder (PTSD) in the perinatal period is estimated at between 3.1% and 7.9% [6–8]. The coexistence of any anxiety disorder with depression is estimated at 9.3% antenatally and 4.2% postnatally [9]. In a US study of pregnant women, women with PTSD, estimated at 7.7%, were five times more likely to also have a major depressive episode, and three times more likely to have generalized anxiety disorder [8].

Perinatal mental health conditions are associated with morbidity and mortality, including risk of developing substance disorders, suicide in severe cases [10–14], and increased adverse infant outcomes, including difficulties with breastfeeding, mother–infant bonding, and parenting [15–18]. FOC has been associated with negative birth experiences [19], and requests for cesarean births [20].

National and international guidelines highlight the perinatal period as a time of significant maternal pressure and rapid infant development, requiring prompt mental health intervention [21–23]. Nonpharmacological interventions are important, given maternal treatment preferences and potential concerns around infant health outcomes [24]. Cognitive behavioral therapy (CBT) is effective in the treatment of perinatal mood and anxiety disorders, and trauma-related disorders [25–29], and is recommended by the United Kingdom's (UK) National Institute for Health and Care Excellence (NICE) for the treatment of antenatal and postnatal mental health [21]. NICE recommendations include CBT-based guided self-help (GSH), for example for mild to moderate depression in the perinatal period, with a growing evidence base for CBT interventions accessed via internet materials, and recognition for a greater efficacy when these interventions are guided [30–33].

Despite the evidence, there are gaps in treatment implementation [34]. The recognition of mental health conditions amongst health professionals has been found to be poor [35, 36], due in part to overlapping symptomatology with pregnancy itself [37]. Furthermore, service providers' may lack cultural competency training and confidence, impacting on their ability to detect perinatal depression, for example in women of African origin [38]. Treatment seeking has been reported to be low, with less than half of new mothers experiencing symptoms seeking treatment [39–41]. Stigma, fears a child may be taken away, language barriers, transportation, childcare difficulties, and a lack of culturally appropriate mental healthcare interventions are amongst the myriad of reasons cited as barriers to mothers seeking a diagnosis and treatment [34, 42–44]. The literature points to findings around reluctance to access to care given cultural expectations that exist for women to be strong and resilient and happy after giving birth [45, 46]. A limited literature exists considering perinatal treatment adherence, with one review of internet-based psychological interventions to treat perinatal depression providing information on dropout rates, ranging from 2.6% to 60.8%, with higher dropouts found for self-guided interventions than those guided by psychological professionals, trained nurses, or midwives [47].

A study of preferences and perceived barriers to treatment for depression during the perinatal period [43], identified themes around adapting CBT to meet the needs of new mothers, which might also improve both treatment uptake and adherence. There are many examples of CBT adapted for the perinatal context across the literature, for example tailoring homework to be relevant to the circumstances and challenges of pregnancy and early postpartum [48]. Other studies have described more extensive adaptations, for example delivering CBT in the home within a wider program of perinatal care, such as home visiting interventions designed for socially isolated mums with low-income [49].

The effectiveness of CBT for perinatal maternal depression, anxiety, and stress has been demonstrated in a review by Li et al. [47], with 79 included studies. The review criteria was broad, examining CBT-only and CBT plus other interventions, for groups, dyads, and individuals, including studies of prevention and treatment, for participants with symptoms below and above a cut-off indicative of diagnostic criteria. Another review of 31 included studies conducted by Pettman et al. [28] has focused on CBT-based interventions for women with a diagnosis of postnatal depression (PND) and/or reporting depression symptomatology within the perinatal period. The reviewers included only CBT-based interventions to reduce clinical heterogeneity, however high heterogeneity was still noted. They found that studies of CBT delivered solely by mental health providers or by mixed providers (for example mental health providers and/or health providers), produced larger effect sizes than CBT delivered by only health or nonspecialist providers. They also found that depression severity at baseline, the inclusion of social and parenting treatment components, and method of CBT delivery did not moderate treatment outcome.

The aim of this review was to examine factors affecting engagement and outcome in studies of individual CBT-only interventions for depression, anxiety, and trauma-related disorders in the perinatal period, to provide new knowledge to improve treatment effectiveness. Previous reviews had examined studies of a wider pool of CBT interventions, including CBT-based interventions and CBT plus other interventions, for individuals, groups, and dyads, with symptoms below and above a cut-off indicative of a diagnostic criteria. Acknowledging the limitations of previous reviews demonstrating high clinical heterogeneity, the review was focused on: randomized controlled trials (RCTs) where more than 70% of women met diagnostic criteria; and CBT that fitted an *a priori* definition and was the active intervention, rather than being part of a wider multicomponent psychosocial intervention.

## 2. Materials and Methods

A protocol was published by the international prospective register, PROSPERO, in November 2022 [50].

### 2.1. Selection Criteria

A table presenting the review eligibility criteria is provided in Table 1.

### 2.2. Search Strategy

Search terms were identified and a systematic search was performed for studies published up to 17^th^ November 2022 via OVID across APA PsycInfo (from 1806), Embase (from 1974), and MEDLINE (from 1946), simultaneously, and via the Cochrane Central Register of Controlled Trials (CENTRAL). The primary search concepts used were 'perinatal period', 'depression, anxiety, and trauma-related disorders', 'psychological therapy', and 'randomized control trial'. Both subject headings and keywords were used in the search strategy. An example search from Medline via Ovid is included as supporting Information (S1). One author (Natalie A. Simon) screened all abstracts and full texts of potentially eligible studies, and a second author (Jenna Evans) double screened 63% of abstracts and 100% of the full texts. Additional studies were retrieved when we checked reference lists of relevant studies. Screening agreement across authors was 98.9%, and we put in place a procedure to involve a third reviewer (Neil P. Roberts) to input where screening disagreements couldn't be resolved, however this was not required.

### 2.3. Methodological Quality Assessment: Risk of Bias

Risk of bias was judged for the following criteria: sequence allocation for randomization; allocation concealment; blinding of assessors; incomplete outcome data; selective outcome reporting; and other notable threats to validity. We included an additional criterion, ‘therapist factors bias'. We did not exclude based on therapist qualifications/training as we thought that this might be an important risk of bias factor. We therefore decided to consider therapist qualifications and training as an additional risk of bias item using criteria described in Table 2.

Data was extracted and synthesized in line with Cochrane Collaboration Guidelines [51], Preferred Reporting Items for Systematic Reviews and Meta-analyses (PRISMA) [52], and synthesis without meta-analyses (SWiM) [53]. The methodological quality of the studies was assessed based on the Cochrane Collaboration's tool for assessing risk of bias (Sterne JAC, [54]. One author (Natalie A. Simon) extracted data and judged risk of bias for every included study, and this was also performed independently for each study by a second author (Jenna Evans, Darcy Bispham, and Ffion Williams). Our procedure to resolve any disagreements with input from a third author (Neil P. Roberts) was not required.

### 2.4. Data Extraction

We prepared a form to systematically extract data. We defined poor treatment outcomes *a priori* as adverse events related to treatment, including an increase in symptomatology posttreatment, and non uptake of, or dropout from treatment, and any other indicators of treatment non acceptability. We defined non uptake as the percentage of women being offered but not taking up treatment, and dropout as the percentage of women dropping out at any time between randomization and the first follow-up assessment. We extracted any other information on treatment engagement and outcome reported in studies, including moderators.

### 2.5. Data Synthesis

There was sufficient data to conduct meta-analyses for dropout. We entered data into the Cochrane Collaboration's Review Manager Web [55]. Categorical outcomes were analyzed as risk ratios (RRs), using 95% confidence intervals. Variability of experimental and comparator interventions, participants, and settings was considered in terms of clinical heterogeneity, as well as considering the *I*^2^ statistic, the chi squared test of heterogeneity, and visual inspection of the forest plots [51]. We planned to pool using a fixed-effect meta-analysis where heterogeneity was low, and with random-effects meta-analysis where heterogeneity was present. We generated funnel plots to assess possible reporting bias since the meta-analyses included more than 10 studies [51]. Other information on treatment engagement and outcome was synthesized and reported as a narrative, following reporting guidelines relevant to reviews incorporating data that are not amenable to meta-analysis [53].

## 3. Results

**Figure 1:**
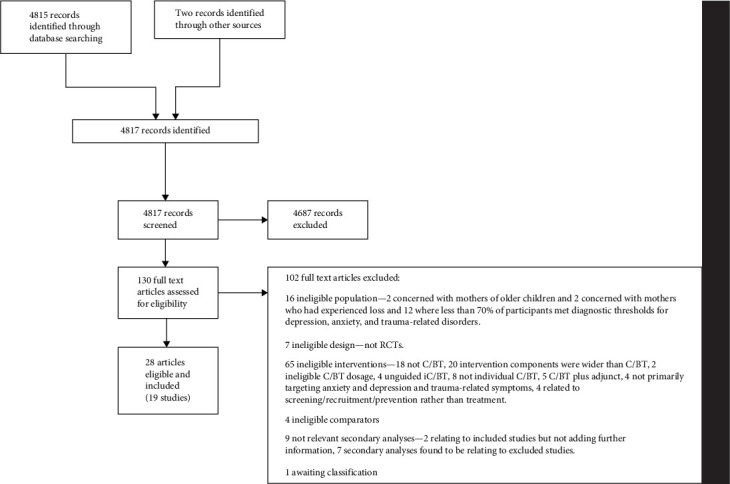
The study selection flow diagram is presented.

The study selection flow illustration is presented as Figure 1. At the initial database search, 4815 records were identified. A further two studies were found from reference list checks. 4817 titles and abstracts were considered, and full texts were obtained for 130 studies, with 102 of these being excluded, including one awaiting classification. Included studies were 28 articles relating to 19 RCTs of 2557 participants.

### 3.1. Study Characteristics

Study characteristics of the 19 included RCTs of CBT are summarized in Table 3. Studies were aimed at a reduction in depression (*k* = 7), major depressive disorder (MDD) (*k* = 2), postpartum depression (PPD) (*k* = 2), PND (*k* = 3), fear of birth (FOB) (*k* = 1), depression and/or anxiety (*k* = 3), and depression, anxiety and/or trauma-related symptoms (*k* = 1). In line with inclusion criteria, all were studies of cognitive and/or behavioral therapy interventions with a therapist or with therapist guidance. Most comparison groups were treatment as usual (TAU) (*k* = 15), the usual or standard treatment as defined by the study authors themselves, and the other comparators were TAU plus information (*k* = 1), psychoeducation (*k* = 1), waitlist (*k* = 1), supportive counseling (*k* = 1). Studies were conducted in Australia (*k* = 3), Canada (*k* = 3), China (*k* = 1), South Korea (*k* = 1), Sweden (*k* = 2), UK (*k* = 5), USA (*k* = 4). Sample sizes ranged from 0 to 100 participants (*k* = 13), 101–400 participants (*k* = 5), and one study of 910 participants. Studies reported baseline symptoms of mild to moderate levels of depression (*k* = 2), moderate depression/anxiety/trauma-related symptoms (*k* = 11), moderate to severe depression/FOB (*k* = 3), and severe depression/anxiety (*k* = 3).

Further information on the interventions that were evaluated in the included studies is provided in Table 4. Studies were of face-to-face cognitive and/or behavioral therapy (C/BT) (*k* = 8), or C/BT via telephone sessions (*k* = 2), and studies of GSH interventions (*k* = 9). Studies of GSH C/BT included therapist guidance via face-to-face sessions and/or telephone contact (*k* = 2), and internet-based GSH interventions with guidance via email and/or telephone (*k* = 8). Intervention duration ranged from 4 to 12 weeks/sessions with a mean of 9.05 weeks/sessions (standard deviation, SD = 2.57). Therapist qualifications, training and supervision varied widely across studies, though most included at least some clinical supervision. Most interventions included the treatment components of psychoeducation, thought monitoring and challenging, management of physical sensations, behavioral activation, and relapse prevention. All but one of the studies reported adaptations made to the interventions to be more relevant for women in the perinatal period. For example, one study included treatment components focusing on child–mother interactions and on changing isolating and avoidance behaviors to break the cycle of loneliness that can come with PPD [80]. Another study included components on thoughts and beliefs around motherhood, cognitive restructuring around motherhood restrictions, interpersonal/social support, and communication skills around conflict support [56].

### 3.2. Methodological Quality of Studies: Risk of Bias

Risk of bias assessments for the included studies are summarized in Table 5. Sequence allocation was judged as low risk in 16 studies and unclear due to limited information in three studies. Allocation concealment was judged to be low risk in 15 studies and unclear in 4 studies. Blinding of the outcome assessor was judged to be low risk in 15 studies, high risk in two studies, and unclear in two studies. Incomplete outcome data was judged to be low risk for 15 studies, high risk for one study, and unclear for three studies. Selective reporting was judged as low risk in 11 studies, high risk in three, and unclear in five studies. Other bias was judged to be high in 13 studies, for example due to the researchers having developed the interventions they were testing, low risk in three, and unclear in three studies. Therapist factors were judged to be low risk in 10, high risk in four, and unclear in five studies.

### 3.3. Adverse Events

Information on adverse events is provided in a table within Table 6. Thirteen studies (68.4%) did not report the presence/absence of adverse events. One study [76], reported an absence of adverse events, and three studies which had described how they would measure adverse events also reported their absence [39, 64, 78]. Two studies reported the presence of adverse events, one [60] reporting a minor event affecting two participants, which was that they felt stressed about not keeping up with the treatment program. Researchers in this study also noted deterioration in one participant in the intervention group and three in the control TAU group, nonsignificant between groups. The other study reporting adverse events reported nine that were not related to mental health or involvement in the trial [79].

### 3.4. Treatment Engagement

Information on treatment engagement, including non uptake and dropout, is provided in Table 6. We defined non uptake as the number of women offered but not taking up treatment, for example being randomized to receive C/BT but not attending or engaging with any sessions or content. There was sufficient information for all but four of the studies. Non uptake ranged from 0% to 66.67% with a mean of 10.53% (SD = 16.60). It was less than 20% in 14 studies, and less than 7% in 10 of these, with a very high rate of 66.67% found for one small study [58], in which only three participants were randomized to receive C/BT, two of whom did not take it up. Four studies (21.1%) reported reasons that invited participants gave for not taking up treatment, which included health concerns [62], and feeling they did not need treatment [58, 69]. Rondung et al. [73, 74] reported that quite a few potential participants declined participation because they did not accept randomization to either intervention, suggesting they would not wish to receive the i/CBT GSH on offer, preferring to receive standard care. Non uptake was mostly unclear for the comparator groups therefore it was not possible to draw meaningful comparisons between treatment arms.

The percentage of women dropping out from C/BT at any time between randomization and the first follow-up was available for 18 studies and ranged from 4.54% to 66.67% with a mean of 19.50% (SD = 17.95). Dropout was less than 20% in 14 studies and less than 10% in seven of these 14 studies. Dropout rates were over 20% in the remaining four studies. Rates were very high in two studies. Two of only three participants randomized to C/BT dropped out in one study, resulting in 66.67% [58], and in another study 281 of 462 (61.8%) participants randomized to i-C/BT GSH dropped out at follow-up [69]. Dropout from the control groups ranged from 0% to 63.84% with a mean of 13.80% (SD = 14.73). Reasons for dropout were not commonly reported, and included being busy and not liking CBT [56], no longer feeling depressed [59], not liking session-by-session questionnaires or childcare difficulties [68], and the inconvenience of the intervention alongside other commitments and symptoms being resolved [79].

**Figure 2:**
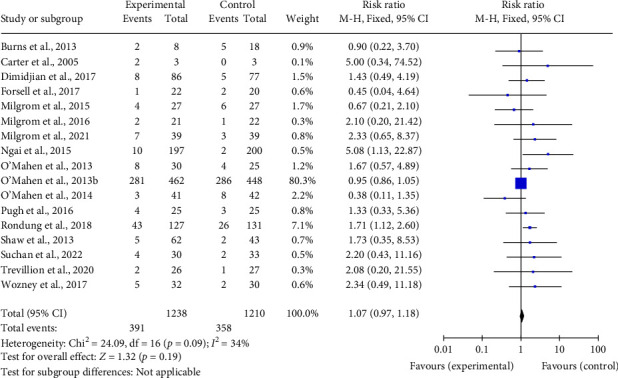
Meta-analyses of dropout of cognitive/behavioral therapy compared with treatment as usual (TAU)/TAU plus information/waitlist.

Results of the meta-analyses of dropout of C/BT compared with TAU/TAU plus information/waitlist, are summarized in a figure presented in Figure 2. There was no evidence of a difference in dropout between groups (*k* = 17; *n* = 2448; RR 1.07; CI 0.97–1.18). It was not possible to conduct meta-analyses of C/BT compared with non-C/BT since only one of these studies provided sufficient information to determine dropout, which was greater for C/BT (20%), compared with psychoeducation (16.67%). Publication bias was not evident on inspection of a funnel plot of the studies included in the meta-analyses.

#### 3.4.1. Sensitivity Analyses

**Figure 3:**
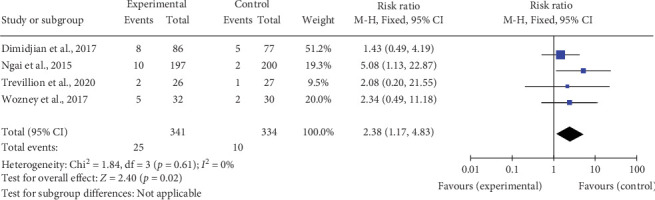
Meta-analyses of dropout of cognitive/behavioral therapy compared with treatment as usual (TAU)/TAU plus information/waitlist for studies where therapists had received minimal training and qualifications in psychological therapies.

**Figure 4:**
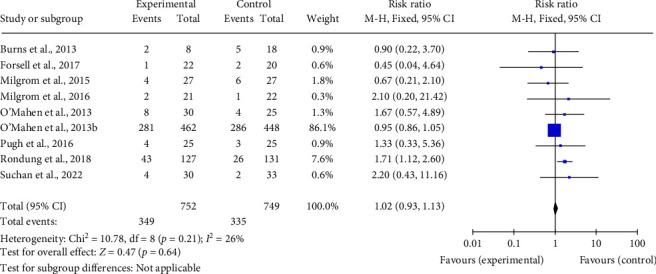
Meta-analyses of dropout of cognitive/behavioral therapy compared with treatment as usual (TAU)/TAU plus information/waitlist for studies where therapists had received at least a moderate level of training and qualifications in psychological therapies.

We conducted post hoc dropout analyses to compare C/BT with TAU/TAU plus information/waitlist for studies where therapist factors were judged as high risk of bias. There was evidence for greater dropout from C/BT compared with the control for studies where therapists had received minimal training in psychological therapies (*k* = 4; *n* = 675; RR 2.38; CI 1.17–4.83) (Figure 3). For studies where therapist factors were judged as low risk of bias, there was no difference in dropout for C/BT compared with the control (*k* = 9; *n* = 1501; RR 1.02; CI 0.93–1.13) (Figure 4).

Every study reported on engagement in various formats (Table 6), with most reporting the percentage of sessions completed, providing *a priori* their definition of completion. Most demonstrated good levels of engagement, with some providing information on how this changed over time, for example O'Mahen et al. [69] reported good initial adherence, reducing over time. Qualitative findings from this study indicated treatment may be improved by personalizing treatment material with examples for mothers to relate to, providing better guidance and support, and addressing individual beliefs about treatment modalities [70]. Rondung et al. [73], reported low adherence to i-C/BT GSH, with fewer than half of participants going on to the second module and less than one-third advancing to the third. O'Mahen et al. [71] reported that women with complex life circumstances required more time to work through the i-C/BT GSH materials and their therapist supporters helped in terms of pacing the treatment content. O'Mahen et al. [68], delivered C/BT in flexible times and locations with intense engagement outreach strategies, noting this was critical to recruitment and retention in their low-income perinatal population. They used a session-by-session measure of adherence and found that barriers to engagement included struggling with the demands of caring for a new baby, child illnesses and pregnancy related pain, housing concerns, and lack of a private, safe home in which to meet.

### 3.5. Studies Reporting an Association Between Treatment Engagement and Treatment Outcome

As shown in Table 6, three studies reported an association between treatment engagement and treatment outcome. Milgrom et al. [62], found a strong dose–response relationship between number of C/BT sessions and amelioration of depression. Another study of C/BT GSH [80], also demonstrated a dose–response relationship between treatment engagement and PPD outcome finding that for every additional session of the intervention received, individuals had a 1.4 times greater chance of showing improvement. They did not however find an association between treatment outcome and treatment duration, nor between treatment outcome and booster sessions. A third study found that both treatment adherence and treatment satisfaction were associated with a reduction in depression scores [68]. Dimidjian et al. [48] examined C/BT reporting an association between self-reported early changes in behavioral activation and later improvements in symptoms of depression, indicating the importance of early engagement and subsequent changes in depressive symptoms.

### 3.6. Factors Associated With Treatment Engagement and Treatment Outcome

As shown in Table 6, two studies found an association between participant demographics and treatment outcome. Hayden et al. [61] found that participant age and race was associated with treatment outcome in their study of C/BT. Shaw et al. [76] examined C/BT for mothers of babies in NICU and found that the mothers' education and household income were nonspecific predictors of the treatment outcome. Less educated mothers and mothers with lower household income showed a greater decline in trauma symptoms irrespective of whether they received C/BT or information/TAU. They also found that the mothers with higher ratings of stress at baseline benefited more from the intervention compared with mothers who had lower stress ratings. They did not however find an association between treatment outcome and other factors of maternal age, current major depressive episode, white/non-white race, Hispanic ethnicity, US born, infant illness severity index, length of NICU stay, and previous trauma history.

Three studies found an association between participant demographics and engagement. O'Mahen et al. 's [71] study of i/CBT GSH with telephone guidance found that women with lower perceived support completed fewer modules, as did women who were working or studying for a degree. They also found that fewer modules were opened by women of lower socioeconomic status and women with poorer work and social adjustment functioning at baseline. They did not however find an association between telephone session adherence and the participant demographics of income, work status, relationship status, academic qualifications, number of children, and ethnicity. Hayden et al. [61] found that participant age and race were associated with attrition. O'Mahen et al. [69], found that women with a higher socioeconomic status were more likely to complete the baseline questionnaire prior to randomization to their study of i-C/BT GSH. Contrastingly, pre-intervention characteristics, including age, relationship status, and education were not found to be associated with attrition in another study of i-C/BT GSH [73]. In this study participants defined as lost to follow-up were no different from those who were not lost to follow-up on the pre-intervention characteristics. In a study that measured participant-rated psychological barriers to help seeking, such as avoidance, these factors were not found to be associated with treatment engagement [68].

Baseline depression was associated with treatment outcome in one study of C/BT [56]. Hayden et al. [61] found that baseline depression was associated with attrition in their study of C/BT, with women reporting higher depression scores and more functional impairment at baseline showing poorer treatment engagement [69]. In contrast, Milgrom et al. [64] did not find an association between attrition and baseline depression in their study of i-C/BT GSH, and Rondung et al. [73] did not find an association between participant FOB scores at screening and being lost to follow-up.

Qualitative findings of a study of i-C/BT with guidance in the form of online messaging [70] suggested a link between hopelessness associated with depression and likelihood of adhering to treatment given that the hopelessness may permeate a belief around getting better. This study also reported views from some participants for a preference for face-to-face care, but also views that the anonymity of internet-based approaches was helpful in terms of stigma, albeit that stigma might still stop mothers from engaging in practical homework activities. Qualitative information from another study of i-C/BT, with text messages and telephone guidance [74], revealed views that the pregnancy itself brought an increased load affecting both family-life, work and the women's health which contributed to a low motivation to complete the i-C/BT intervention, though a subsample of mothers were motivated towards using i-C/BT.

### 3.7. Treatment Satisfaction and Therapeutic Alliance

As shown in Table 6, treatment satisfaction was reported at high levels across the 10 studies measuring it via a range of measures. Forsell et al. [60] reported good i-C/BT GSH treatment credibility, indicating how logical and convincing the treatment is, according to the treatment credibility scale (TSQ) [81]. They also reported satisfaction according to the Client Satisfaction Questionnaire-8 (CSQ-8) [82], with most participants considering the pregnancy-adaptation of the treatment to be helpful and important. O'Mahen et al. [69], had a low response rate (22%) to their acceptability questions, but responses revealed acceptability of i-C/BT GSH. Participants' views of the applicability of C/BT treatment materials was found to be associated with treatment satisfaction in one study [68]. Only three of 19 studies (15.8%) measured therapeutic alliance, with high levels reported across these studies. In a study of i-C/BT GSH, most participants reported enjoying communicating with their therapist [72]. In another study of C/BT GSH [79], therapists demonstrated good therapeutic alliance according to the interpersonal effectiveness item of the Cognitive Therapy Scale Revised (CTSR) [79, 83]. Participants in a study of i-C/BT [78] expressed high levels of alliance with therapists according to the Working Alliance Inventory (WAI) [84] Greenberg, 1989).

## 4. Discussion

This review examined factors affecting engagement and outcome in studies that delivered individual CBT for depression, anxiety, and trauma-related disorders in the unique perinatal context. We reviewed 28 studies of 19 RCTs with 2557 women. There were no major treatment-related adverse events reported. Engagement was associated with outcome in three studies, two of which demonstrated a dose–response relationship. Treatment satisfaction was high for most studies and therapeutic alliance was high for the three studies that measured this construct. Few studies examined factors associated with treatment outcome, two reported participant age and race, and another reported baseline depression to be significantly associated with treatment outcome. Inconsistent and absent reporting of factors associated with treatment outcome is also evident elsewhere in the literature [85], despite a vital need to understand the reasons why people may or may not be benefitting from treatment. This is particularly pertinent in this population given the unique context of the perinatal period.

All included RCTs reported mostly good levels of CBT engagement, across a range of quantitative indicators and qualitative information. Meta analysis demonstrated that there was no difference in dropout between CBT and TAU/TAU plus information/waitlist across 17 studies. This is in line with findings by Cuipjers et al. [86], indicating a nondifferential dropout rate between psychotherapy treatment for perinatal depression, including CBT, and control groups across 43 comparisons. We estimated dropout from CBT to be 19.50% across 18 studies (SD = 17.95), and less than 10% in seven of 18 RCTs, indicating good adherence. These findings are comparable to limited findings elsewhere in the literature. For example, a review of self-help and guided digital mental health programs for women in the perinatal period reported dropout rates between 10% and 87% [87]. More broadly, dropout rates of 26.2% have been reported across CBT studies with child and adult populations [88], 17.5% across RCTs of psychotherapy for depression [89], and 16% across RCTs of psychological therapies for PTSD [90]. Participant demographics, including age, race, perceived social support, and whether they were working or studying for a degree were associated with treatment engagement, albeit inconsistently. For example, women with lower perceived social support completed fewer treatment modules in O'Mahen et al. 's RCT [71].

Qualitative findings included a mixture of views around treatment, including a lack of motivation to engage given the increased challenges and responsibilities brought about by pregnancy and motherhood, in line with literature reporting that women in the perinatal period are presented with a unique set of barriers to treatment [42]. The literature reports a mixture of views around mental health treatment, not limited to the perinatal context, for example a systematic review of barriers and facilitators to mental health service utilization in adult trauma survivors, which reported many factors, including lack of time, negative experiences with professional help, and prioritizing the needs of others [91]. The present review findings highlighted the helpfulness of treatment content made relevant to the perinatal context and guidance and support. Greater dropout from CBT compared to TAU/TAU plus information/waitlist was found across four studies where the therapist/coach guiding the CBT interventions had a minimal level of psychological therapy training and qualifications. These findings align with research demonstrating the effectiveness of guided CBT interventions [31], particularly where guidance is provided by a mental health provider compared with a non specialist provider [28, 47].

### 4.1. Strengths and Limitations

This was the first review of factors associated with treatment outcome for women in the perinatal period receiving individual CBT for depression, anxiety, and trauma-related disorders. We restricted to studies of the rigorous RCT design, examining guided interventions of CBT fitting our definition, with more than 70% of participants meeting diagnostic criteria. This enabled meaningful comparisons to be made across the included studies and populations. Studies were however mostly conducted in Western industrialized countries therefore caution must be taken when generalizing the findings to perinatal populations in other non-Western, and especially low- and middle-income countries. Examining secondary analysis papers allowed for further exploration of a myriad of matters related to treatment outcome and engagement, including qualitative findings.

Methodological challenges were evident. No cut-off date was applied in the search strategy, therefore a proportion of the records to be considered were of none or little relevance to the focused research question concerning modernized individual CBT interventions. There were a range of other challenges which are commonly found across the literature. Many included studies were of small sample sizes which may have been underpowered to detect factors affecting engagement and outcome. We noted other risks of bias in the included studies, limiting our confidence in the quality of some. We examined adverse events, including an increase in symptomatology posttreatment, however we did not have individual level data and adverse event reporting was absent for 13 of the 19 included studies (68.4%), a limitation noted in the wider psychological literature [92]. We examined treatment non uptake and dropout, however reasons were inconsistently reported and dropout interpretation may be hampered without consistent reporting of reasons for discontinuing treatment [93]. Eligible comparison groups included TAU, which was usual or standard treatment as defined by the study authors themselves. There was considerable variation in reporting TAU across studies and where it was reported there was great variability in terms of treatments and supports accessed by individuals randomized to TAU. The challenge of disentangling the potential confounding effects of additional interventions and support systems is another commonly occurring methodological challenge in systematic reviews [94]. To illustrate, TAU or usual care may include a combination of interventions, including pharmacological and psychological therapy, depending on the needs, preferences, and circumstances of an individual patient and their developing fetus or newborn child. Treatment or usual care in the perinatal period would depend on case management, balancing patient choice, informed by potential risks and benefits, for example psychotropic medication use and breastfeeding goals. Participant ethnicity, culture and religion were not consistently reported, limiting our ability to consider these important demographics which may be playing a role in perinatal beliefs, interpretation of symptoms, and CBT engagement [95].

### 4.2. Research and Clinical Implications

Most of the included studies were concerned with CBT for depression. More research is needed examining CBT for anxiety and trauma-related disorders in the perinatal period. Future research in the area should examine and report on treatment engagement, outcome, and moderators, including examining individual level data, to optimize analyses and interpretation. Treatment engagement reporting should be consistent and in line with guidelines, for example online intervention reporting guidelines [96], not least given the difficulty found when measuring CBT GSH engagement [97]. Routine measurement and reporting of treatment satisfaction and therapeutic alliance would help in understanding the optimum levels of guidance and support required for engagement and recovery. Standardized measurement and reporting would allow more meaningful comparisons across studies to be made. This might include consistently measuring the relationship between engagement and outcome and moderators. Standardized reporting around TAU would also be beneficial. TAU was the most common comparator group in the present study, however there was wide variability in TAU reporting. Where it was reported, there was within study variability of interventions, including pharmacological approaches. Case management in perinatal mental health considers NICE guidelines [24], of patient choice, with a comprehensive evidence-based approach exploring a combination of psychological and pharmacological treatments, for example combining CBT for depression with antidepressant medication. The risks and benefits of pharmacotherapy and psychotherapy approaches should be carefully considered by clinicians and patients. Both approaches have been shown to be comparatively effective in treating a range of disorders in adult mental health [98]. Further research and exploration is required, with a focus on perinatal mental health, to understand the risks and benefits of approaches used independently and when combined. There are many differences in the methods used across trials of both approaches, making comparisons difficult.

The findings of this review highlighted an important relationship between CBT treatment engagement and outcome, which has clinical implications. Most studies included CBT adapted to the perinatal context, and findings indicated this aspect to be important in terms of optimizing engagement, given the unique challenges faced by women in the perinatal period. Findings also implicate therapeutic guidance for treatment engagement, with provision preferably from therapists or coaches who have more than the minimal amount of psychological therapy training and qualifications. Careful individual-level formulation should be taken by clinicians in collaboration with a woman in the perinatal period, to make a shared decision around CBT treatment. This would include considering motivation towards treatment, and whether there would be a preference for face-to-face CBT, or whether CBT GSH may be suitable, which may be an empowering and cost-effective alternative offer depending on an individual's circumstances [99, 100]. Clinicians and patients should consider the cultural appropriateness of CBT interventions, and whether culturally adapted interventions are available to enhance engagement and effectiveness [95]. Clinicians may wish to adapt and personalize interventions, for example offering flexibility around pace and time allocated to treatment components, to suit needs and preferences and cultural and religious values. Given the standard RCT design often lacks the flexibility to personalize interventions, practice-based evidence may be particularly helpful. Practice-based research is required to examine how adaptations may or may not be impacting on engagement and outcome, for continuous evaluation and improvement.

## 5. Conclusions

This review revealed an important relationship between treatment engagement and outcome for women receiving individual CBT for depression, anxiety, and trauma-related conditions in the perinatal period. There was no difference in dropout for studies of CBT compared to TAU, but there was greater dropout for CBT across four studies where the guiding therapist/coach had a minimal level of psychological therapy training and qualifications. Findings suggest that treatment engagement and outcome may be improved when CBT is adapted to meet the unique needs faced by women in the perinatal period, and when it is provided by therapists with at least a moderate amount of psychological therapy training and qualifications. Future work is therefore required to examine standards in the training of therapists and coaches who would be delivering CBT in the context of medical care. Working to ensure at least a moderate level of psychological training received, to optimize CBT for new and expecting mothers. Work is also required to examine and report on treatment engagement, outcome, and moderators, including examining individual level data, to optimize analyses and interpretation. Routine and consistent measurement and reporting of factors related to engagement and outcome are required.

## Figures and Tables

**Table 1:** The review eligibility criteria.

We included:	We excluded:
Randomized controlled trials published in English (randomized crossover trials and cluster-randomized trials);	Studies where less than 70% of participants met diagnostic criteria for anxiety, depression, or trauma-related disorders according to Diagnostic and Statistical Manual of Mental Disorders (DSM), and International Classification of Diseases (ICD) criteria, via clinical interview or validated self-report questionnaire (where self-report questionnaires were used, we estimated clinical caseness using data provided in the study, and established cut-offs for particular measures);
Studies of women aged over 16 of any ethnicity in the perinatal period (defined as pregnant or up to 2 years postnatal at the time of the baseline assessment);	Studies where the intervention was focused around the loss of a child;
Studies where individual cognitive behavioral therapy (CBT) was the active intervention, described in sufficient detail and meeting our definition of CBT (defined as established models of cognitive and/or behavioral therapy, with cognitive therapy built around the concept that the way we think affects how we feel emotionally and how we seek to behave, and behavioral therapy being based on changing behavior, for example behavioral activation or exposure-based work), delivered with or without treatment as usual;	Studies of brief interventions (which we defined as sessions lasting less than half an hour or of a duration of three or fewer sessions);
Studies where a reduction in anxiety, depression, or trauma-related symptoms was the primary aim of the intervention (including where anxiety, depression, or trauma-related disorders were comorbid disorders);	Studies where CBT for the targeted problem was one part of a wider multicomponent psychosocial intervention;
Studies of CBT delivered face-to-face or remotely, including guided self-help, such as internet-based programs with guidance either in person, by telephone or by email/messenger, (automated instant messaging was not included);	Studies testing psychoeducation, mindfulness-based interventions and EMDR.
Studies comparing CBT with waitlist, repeated assessment, treatment as usual, face-to-face and internet-based psychological therapy which was non-CBT-based (for example including Eye Movement Desensitization and Reprocessing (EMDR), supportive therapy, nondirective counseling, psychodynamic therapy, and present-centered therapy).	

**Table 2:** Additional therapist qualifications and training risk of bias item.

Low risk of bias	High risk of bias	Unclear
Studies where the therapist had at least a moderate level of psychological therapy qualifications/training, for example a clinical psychologist a cognitive behavioral therapy (CBT) therapist a psychological wellbeing practitioner with CBT training, or in the case of the United States of America or Canada, a social worker with CBT training.	Studies where the therapist had minimal psychological therapy qualifications/training, for example an undergraduate psychology student with no further training, or a healthcare professional with no previous therapeutic experience or only a few days of CBT training.	Studies where the therapist qualifications and training are not reported, or the information is unclear.

**Table 3:** Included study characteristics.

Study	*N*	Country	Recruitment	Condition	Method of diagnosing the condition	Intervention	Control	Baseline severity	Mean age (and standard deviation, SD)	Ethnicity (%)	% in a relationship	% University education
Burns et al. [56], Netsi et al. [57]	36	UK	Via routine midwife contact referral	Depression	CIS-R	C/BT	TAU	Moderate	Intervention 28.2 (SD = 5.0) Control 30.1 (SD = 6.2)	Intervention white 72.2% and Control white 94.4%	Intervention 72.2% and Control 55.6%	Reported as ‘O' level equivalent or above, as intervention 83.3% Control 88.9%
Carter et al. [58]	6	UK	Via routine maternity clinics	MDD	SCID	C/BT	TAU	Moderate	Not reported	Not reported	Not reported	Not reported
Cho et al. [59]	27	South Korea	Via routine maternity clinics	Depression	SCID-I	C/BT	PsychoEd	Moderate	Intervention 30.3 (SD = 4.1) and Control 28.8 (SD = 2.7)	Not reported	Not reported, however, the intervention was concerned with improving marital satisfaction therefore might assume all married	Intervention 50% Control 40%
Dimidjian et al. [48]	163	USA	Referral via obstetric care providers, or via self-referral following healthcare appointment	Depression	Self-reported PDSQ	C/BT BA plus TAU	TAU	Moderate	28.75 (5.67)	White 58.28%, Black 27.61%, Asian 4.29%, and other 9.82%	69.94%	13.50%
Forsel et al. [60]	42	Sweden	Self-referral via web-based and public advertisements and via healthcare signposting	Depression and anxiety	SCID-I	i-C/BT GSH	TAU with option to receive i-C/BT at a later date	Moderate	Intervention 31.2 (SD = 3.7) and Control 30.8 (SD = 5.3)	Not reported	Intervention 96% and Control 100%	Intervention 86% Control 60%
Hayden et al. [61]	34	USA	During health seeking appointments at prenatal care clinics	MDD	Diagnostic Interview Schedule	C/BT	Supportive counseling	Moderate	Intervention 31.5 (SD = 6.1) and Control 30.7 (SD = 5.0)	Only reported as follows: intervention minority participants 35.3% and Control 70.6%	Not reported	Not reported
Milgrom et al. [62, 63]	54	Australia	Via routine maternity clinic referral and via other healthcare professional referrals	Depression and anxiety	SCID	C/BT	TAU	Severe	Intervention 32.79 (SD = 5.97) and Control 30.78 (SD = 5.86)	Reported those born in Australia as intervention 74% Control 81.5%	Intervention 92.6%, Control 85.2%	Intervention 48.1%, Control 51.8%
Edge [39]	43	Australia	Self-referral via web-based advertisements and via healthcare signposting	Depression	SCID-IV	i-C/BT GSH	TAU	Moderate	Intervention 31.7 (SD = 4.6) and Control 31.5 (SD = 4.3)	Only reported that 91% born in Australia	88.5%	49%
Milgrom et al. [64]^a^	116	Australia	Self-referral via web-based and public advertisements, and via maternal and child health centers	PND	SCID-IV	i-C/BT GSH	TAU	Moderate to severe	Intervention 30.8 (SD = 4.3) and Control 31.9 (SD = 4.2)	Not reported	Intervention 100% Control group 92%	Intervention 23% Control 24%
Ngai et al. [17, 65–67], Netsi et al. [57]	397	China	During postnatal hospital unit admissions	Depression	Self-report EPDS	C/BT	TAU	Mild to moderate	Intervention 31.1 (SD = 3.8) and Control 30.4 (SD = 4.4)	Not reported	100%	Intervention 56.9%, Control 56%
O'Mahen et al. [68]	55	USA	Via obstetric clinic referral	Depression	SCID-I	C/BT	TAU	Moderate	Intervention 27.40 (SD = 5.32) Control 26.62 (SD = 6.01)	Intervention African American 16%, white 10%, Asian 3%, Other 1% Control African American 16%, White 7%, Asian 1%, Other 1%	Intervention partnered 70% Control 64%	Intervention 36.6% Control 16%
O'Mahen et al. [69, 70]	910	UK	Self-referral via web-based advertisements	PND	EPDS	i-C/BT GSH	TAU	Moderate to severe	Interventionn32.3 (SD = 4.7) Control 32.2 (SD = 5.7)	Not reported	93.5%	44.4%
O'Mahen et al. [71]	83	UK	Self-referral via web-based advertisements	PND	CIS-R	i-C/BT GSH	TAU	Severe	Not reported	Intervention white British 92.6%, Asian 2.4%, Other 4.8% Control white British 92.9%, Mixed white/African/Caribbean 4.8%, African 2.4%	Intervention 92.6% Control 90.5%	Intervention 56.1% Control 50%
Pugh et al. [72]	50	Canada	Self-referral via web-based advertisements and via healthcare signposting	PPD	MINI	i-C/BT GSH	Waitlist	Moderate	Not reported	Intervention Caucasian 92%, Other 8% Control Caucasian 100%	Intervention 92% Control 78%	Intervention 59% Control 70%
Rondung et al. [73], Baylis et al. [74], Hildingsson and Rubertsson [75]	258	Sweden	Via routine maternity clinics	FOB	FOB scale	i-C/BT GSH	TAU	Moderate to severe	<25 14.3%, 25–35 72.1%, >35 13.6%	Reported country of birth as Sweden 86.8% and other country 13.2%	Reported living with partner as 94.2%	53.5%
Shaw et al. [76, 77]	105	USA	Parents of hospitalized infants within NICU wards were approached	Depression, anxiety, and trauma-related symptoms	Davidson trauma scale for PTSD, MINI for anxiety and depression	C/BT	Information/TAU	Moderate	Intervention 33.76 (SD = 6.25) Control 30.70 (SD = 5.50)	Intervention white 67.7%, Hispanic 25.8% Control white 51.2%, Hispanic 32.6%	Intervention 93.5% Control 100%	Intervention 69.4% Control 55.8%
Suchan et al. [78]	63	Canada	Self-referral via web-based advertisements and via healthcare referral	Depression and/or anxiety	Self-report EPDS, GAD-7, PHQ-9	i-C/BT GSH	TAU	Moderate	30.83 (SD = 4.29)	85% white, 8% aboriginal, 7% other	96%	57%
Trevillion et al. [79]	53	UK	Via participation in a related study, or via midwife referral, or self-referral through poster adverts	Depression	Clinical interview based on DSM-IV (name not reported)	C/BT GSH plus TAU	TAU	Mild to moderate	Intervention < 25 11.54%, 25–29 19.23%, 30–39 69.23%, 40 + 0% Control < 25 7.41%, 25–29 11.11%, 30–39 66.67%, 40 + 14.81%	Intervention White 69.23%, Black 26.92%, Asian 0%, Mixed/Other 3.85%; Control White 69.96%, Black 25.93%, Asian 3.70%, Mixed/Other 7.41%	Intervention 73.08% and Control 66.67%	Intervention 61.54% and Control 55.56%
Wozney et al. [80]	62	Canada	Self-referral via web-based, and public advertisements and via healthcare provider referral	PPD	SCID-I	C/BT GSH	TAU	Severe	Intervention 28.0 (SD = 3.9) and Control 29.9 (SD = 5.6)	Not reported	Intervention 25% and Control 25%	Intervention 12% and Control 15%

^a^This study [25] included a third comparison group, which was face-to-face cognitive/behavioral therapy but data from this ineligible comparison group was not included in this review. CIS-R, a self-administered computerized interview and gives an ICD-10 diagnosis of mild, moderate or severe depression.

Abbreviations: BA, behavioral activation; C/BT, cognitive/behavioral therapy; DSM, diagnostic and statistical manual for mental disorders; EPDS, Edinburgh postnatal depression scale; FOB, fear of birth; GAD-7 - generalized anxiety disorder assessment; GSH, guided self-help; i-C/BT, internet-based cognitive/behavioral therapy; MDD, major depressive disorder; MINI, mini international neuropsychiatric interview; NICU, neonatal intensive care unit; PDSQ, psychiatric diagnostic screening questionnaire; PHQ-9, patient health questionnaire; PND, postnatal depression; PPD, postpartum depression; PsychoEd, psycho education.PTSD, post-traumatic stress disorder; SCID, structured clinical interview for DSM; TAU, treatment as usual.

**Table 4:** Treatment components of included studies.

Study	Intervention	Intervention name	Treatment duration/dosage	Therapist qualifications and training and supervision	Psychoeducation on the condition and the link between thoughts, physical sensations, and behaviors	Thought monitoring and challenging/restructuring	Managing physical sensations, for example grounding and breathwork	Behavioral activation and/or graded exposure, for example to manage mood and increase pleasurable activities	Future planning and relapse prevention	Adaptations or additional components specific to the perinatal period for example disrupted sleep, isolation, role transitions, physical challenges associated with pregnancy/childbirth/breastfeeding
Burns et al. [56], Netsi et al. [57]	C/BT in person face-to-face	CBT for antenatal depression	Up to 12 sessions	CBT therapists with masters/doctoral experience in CBT, with training and weekly supervision form a clinical psychologist. Competence assessed using the revised cognitive therapy scale (CTS-R).	Included	Included	Included	Included	Included	Treatment provided in the individual's home, with all but one of the participants accepting this invitation. Thoughts and beliefs around motherhood, including cultural beliefs, BA in relation to cognitive restructuring around restrictions around motherhood, interpersonal/social support, and communication skills around conflict support.
Carter et al. [58]	C/BT in-person face-to-face	C/BT	12 weekly sessions	Not reported	Included	Included	Included	Included	Included	Treatment could be provided in the mother's home.
Cho et al. [59]	C/BT in person face-to-face	CBT	9 intervention sessions delivered as 1-h sessions that were biweekly	The therapist delivering CBT was a licensed clinical psychologist and they received supervision from a licensed supervisor for CBT	Included	Included	Included	Included	Unclear	Enhancing the marital relationship through the review and modification of dysfunctional cognition in the relationship.
Dimidjian et al. [48]	C/BT BA in person face-to-face (or telephone if preferred) plus usual care	BA tailored for depressed pregnant women	10 sessions but encouraged to pace flexibly to accommodate mother's scheduling demands	Nurse midwives, practitioners, and an occupational therapist attended training in BA and clinical supervision.	Included	Unclear	Unclear	Included	Unclear	Treatment could be in the mother's home, clinic or by telephone. Clinical examples and assignments were tailored to the circumstances and challenges of pregnancy and early postpartum.
Forsel et al. [60]	i-C/BT GSH with online message contact with therapist	i-C/BT	10 sessions with written messages from therapists	CBT-trained and regularly supervised therapist (supervision with psychologist, obstetricians, and psychiatrist)	Included	Included	Included	Unclear	Unclear	Pregnancy, including the relation between pregnancy and depression and a module on relationships.
Hayden et al. [61]	C/BT in-person face-to-face	C/BT	10 weekly sessions	Administered by licensed clinical social workers who received specific CBT training and supervision from a licensed clinical psychologist certified as a CBT supervisor	Unclear	Included	Unclear	Unclear	Unclear	Unclear
Milgrom et al. [62, 63]	C/BT in person face-to-face	Beating the blues before birth CBT intervention	8 treatment sessions of 1 h each per week	Psychologists with a background in CBT were trained in the program and delivered the intervention.	Included	Included	Included	Included	Included	Refinement to accommodate needs of postnatal women included introducing BA before cognitive strategies, addressing partner and infant issues, and issues around transition to parenthood. Partners included for one of the sessions.
Milgrom et al. [39]	i-C/BT GSH with telephone sessions with therapist	MumMoodBuster program	6 sessions (at own pace but recommended one session per week)	Graduate psychology trainees, clinical psychologists, health psychologists, trained and supervised by senior psychologists.	Unclear	Included	Unclear	Included	Included	Participants and their partners were granted access to a library of resources, including parenting support and information on PND.
Milgrom et al. [64]	i-C/BT GSH with telephone sessions with therapist	MumMoodBuster program	6 sessions (at own pace but recommended one session per week)	Unclear	Included	Included	Unclear	Included	Unclear	Participants and their partners were granted access to a library of resources, including parenting support and information on PND.
Ngai et al. [17, 65–67], Netsi et al. [57]	C/BT via telephone	Telephone-based CBT	5 weeks	Midwife with 20 h of CBT training with supervision and fidelity control	Included	Included	Unclear	Included	Included	Goal setting in relation to the maternal role was emphasized as were coping skills and decision-making in relation to practical issues of childcare and common neonatal problems.
O'Mahen et al. [68]	C/BT in-person face-to-face	CBT for perinatal depression adapted for women with low incomes	12 weeks	Masters and doctoral level social workers and psychologists with experience in CBT and/or treatment for perinatal depression. Clinical training and supervision.	Included	Included as an additional module for individuals with depressive cognitions or interpersonal difficulties	Unclear	Included	Unclear	Additional resources on perinatal specific materials and skills, including sleep hygiene. Option for treatment in the home, with 73% of sessions happening in the home. Additional module also on interpersonal support for those requiring this.
O'Mahen et al. [69, 70]	i-C/BT GSH with online message contact with therapist	Postnatal iBA	11 sessions to be completed weekly or within 15 weeks	Therapists were either a specialist health visitor or clinical psychologist	Included	Not included, authors give rationale for, not including cognitive restructuring	Unclear	Included	Included	Treatment focused on helping mothers achieve a balance in valued activities in the context of competing and unpredictable demands. Signposting to resources on parenting and mother-related resources and activities and to a supervised web forum/chat room.
O'Mahen et al. [71]	i-C/BT GSH with telephone contact with therapist	NetmumsHWD which built on the postnatal iBA noted above, adapted to be modular and with increased support	12 weeks	Mental health workers with undergraduate degrees with 1 year IAPT psychological therapies clinical qualification. Training and supervision with a clinical psychologist.	Included	Included as an optional module	Unclear	Included	Included	Additional modules focusing on being a good enough mother, sleep, and other perinatal specific content. Signposting to resources and a supervised web forum.
Pugh et al. [72]	i-C/BT GSH with email contact with therapist (telephone contact if required)	Maternal depression online, developed by adapting TA-ICBT for depression	7 modules recommended one per week	Facilitated by doctoral students in clinical psychology with training and supervision from a registered psychologist	Included	Included	Included	Included	Unclear	Incorporated adaptations relevant to mothers of young infants, though no specific details provided.
Rondung et al. [73], Baylis et al. [74], Hildingsson and Rubertsson [75]	i-C/BT GSH with text and telephone contact with therapist	i-C/BT, inspired by the unified protocol for treatment of emotional disorders (UP), a face-to-face protocol, designed for applicability to all anxiety and unipolar mood disorders	8 sessions recommended weekly	Guided by licensed clinical psychologists	Included	Included	Included	Included	Included	Adapted for the population regarding the content and order of the psychoeducation elements and FOB-specific examples. Additional module for postpartum follow-up focusing on childbirth in retrospect.
Shaw et al. [76, 77]	C/BT in person face-to-face	Manualised intervention for NICU parents	3 – 4 sessions with one or two 45-to 55- minute sessions administered weekly	Clinical psychology graduate program students and a NICU social worker with training and supervision.	Included	Included	Included	Included	Included	Two additional sessions, adapted with permission from the creating opportunities for parent empowerment program to enhance the mother–infant relationship, also known as infant redefinition.
Suchan, et al. [78]	i-C/BT GSH with email contact with therapist (telephone contact if required)	Wellbeing course for new moms	8 weeks	Master-level, registered social worker trained in the provision of i-CBT GSH	Included	Included	Included	Included	Included	Case stories were used that were relevant to new mothers and additional resources were available detailing common struggles experienced by new mums.
Trevillion et al. [79]	C/BT GSH workbook with either face-to-face or telephone guidance, plus usual care	Modified GSH, with usual care, for antenatal depression	An initial session plus eight 30-minute sessions and a final check-in session 6–8 weeks postdelivery	Psychological wellbeing practitioners experienced in delivering treatments based on GSH manuals, with clinical training and supervision	Included	Included	Unclear	Included	Included	Managing relationships in the perinatal period, including increasing social support. The development of maternal–fetal attachment and reflecting on how we learn to be parents. Preparing for parenthood.
Wozney et al. [80]	C/BT GSH with telephone sessions with therapist	The strongest families: managing our mood (MOM)	12 sessions	Coaches were paraprofessionals who completed MOM coach training and received 1:1 supervision from an expert clinician throughout study	Included	Included	Included	Included	Included	A focus on child–mother interactions and on changing isolating and avoidance behaviors to break the cycle of loneliness that can come with postpartum depression.

*Note:* CTS-R, revised cognitive therapy scale; TA-ICBT for depression, therapist-assisted internet-delivered CBT.

Abbreviations: BA, behavioral activation; C/BT, cognitive/behavioral therapy; GSH, guided self-help; i-C/BT, internet-based cognitive/behavioral therapy; IAPTs, improving access to psychological therapies; IBA, internet-based behavioral activation; NetmumsHWD, netmums helping with depression; NICU, neonatal intensive care unit; PND, postnatal depression.

**Table 5:** Risk of bias.

Study	Random sequence generation	Allocation concealment	Blinding of outcome assessment	Incomplete outcome data	Selective reporting	Other bias	Therapist factors bias
Burns et al. [56]	Low risk	Low risk	Low risk	Low risk	Low risk	Unclear	Low risk
Carter et al. [58]	Unclear	Unclear	Unclear	High risk	Unclear	High risk	Unclear
Cho et al. [59]	Unclear	Unclear	Low risk	Low risk	Unclear	Low risk	Low risk
Dimidjian et al. [48]	Low risk	Low risk	Low risk	Low risk	Low risk	Unclear	High risk
Forsel et al. [60]	Low risk	Low risk	High risk	Low risk	Low risk	Unclear	Unclear
Hayden et al. [61]	Low risk	Unclear	Low risk	Unclear	High risk	High risk	Low risk
Milgrom et al. [62]	Low risk	Low risk	Low risk	Low risk	Low risk	High risk	Low risk
Milgrom et al. [39]	Low risk	Low risk	High risk	Low risk	High risk	High risk	Low risk
Milgrom et al. [64]	Low risk	Low risk	Low risk	Low risk	Low risk	High risk	Unclear
Ngai et al. [17]	Low risk	Low risk	Low risk	Low risk	Low risk	Low risk	High risk
O'Mahen et al. [68]	Low risk	Low risk	Low risk	Low risk	Low risk	Low risk	Low risk
O'Mahen et al. [69]	Low risk	Low risk	Low risk	Unclear	Unclear	High risk	Low risk
O'Mahen et al. [71]	Low risk	Low risk	Unclear	Low risk	Unclear	High risk	Unclear
Pugh et al. [72]	Low risk	Low risk	Low risk	Low risk	Low risk	High risk	Low risk
Rondung et al. [73]	Unclear	Unclear	Low risk	Low risk	High risk	High risk	Low risk
Shaw et al. [76]	Low risk	Low risk	Low risk	Low risk	Low risk	High risk	Unclear
Suchan et al. [78]	Low risk	Low risk	Low risk	Unclear	Low risk	High risk	Low risk
Trevillion et al. [79]	Low risk	Low risk	Low risk	Low risk	Low risk	High risk	High risk
Wozney et al. [80]	Low risk	Low risk	Low risk	Low risk	Unclear	High risk	High risk

*Note:* High risk would be where the therapist has minimal psychological therapy qualifications/training, for example someone with an undergraduate degree in psychology with no further training, or a midwife with perhaps a few days of training in CBT but no previous therapeutic experience. Low risk would be a therapist with at least moderate psychological therapy qualifications/training, for example a clinical psychologist or a CBT therapist or a psychological wellbeing practitioner with CBT training. Please note that social workers in America have roughly equivalent training and experience in psychological therapies as psychological practitioners in the UK.

**Table 6:** Factors associated with treatment engagement and outcome.

Study	Intervention and comparator	Adverse events	Treatment nonuptake	Treatment dropout/attrition	Treatment engagement, satisfaction, and therapeutic alliance	Is there an association between treatment adherence and treatment outcome?	Factors associated with treatment engagement and treatment outcome	Other findings relevant to treatment engagement and treatment outcome
Burns et al. [56]	C/BT vs. routine clinical care	Not reported	Unclear	Intervention 2 of 18 (11.11%) Control 5 of 18 (27.78%) Reasons given for withdrawal from CBT included being too busy and disliking CBT.	Good engagement, completion rate of 72%	Not reported	(−) Baseline depression score and improvements in depression scores. (+) Improvement in depressive symptoms and easier infant temperament and shorter nocturnal sleep duration, (*n* = 25).	Not reported
Carter et al. [58]	C/BT vs. routine clinical care	Not reported	Intervention 66.67% Control 0%. Reasons for nonuptake of C/BT treatment were reported as feeling they did not need treatment.	Intervention 2 of 3 (66.67%) Control 0 of 3 (0%)	Good engagement for the one individual who received, 100% completion rate.	Not reported	(+) Women who were more symptomatic on the EPDS were more likely to agree to be contacted to be considered for participation in the study (only six individuals were randomized).	Not reported
Cho et al. [59]	C/BT vs. psychoeducation	Not reported	Intervention 0% Control 0%	Intervention 3 of 15 (20%) Control 2 of 12 (16.67%), with reasons, including premature delivery and no longer feeling depressed	Good engagement, 80% completion rate.	Not reported	Not reported	The intervention included the review and modification of dysfunctional cognition in the marital relationship of the parents. This improved through treatment.
Dimidjian et al. [48]	C/BT BA plus TAU care vs. TAU	Not reported	Intervention 6.98% Control unclear	Intervention 8 of 86 (9.30%) Control 5 of 77 (6.49%)	Good engagement, two-thirds of participants completing five or more sessions (M 6.43; SD = 3.64). High degree of satisfaction on the CSQ-8 (M 27.76; SD = 3.83).	Not reported	Not reported	Early change in self-reported putative targets was found to predict later improvement in symptoms of depression (NB - the causal relationship cannot be inferred through the methodology, which was underpowered).
Forsel et al. [60]	i-C/BT GSH vs. TAU with option to receive i-C/BT at a later date	Two patients reported one minor adverse event, which was that they felt stressed about not keeping up with the treatment program. Deterioration (considered as 4 points or more increase on the primary outcome MADRS-S) was observed in three participants in TAU and one participant in i-CBT but the difference was not significant.	Intervention 4% Control unclear	Intervention 1 of 22 (4.54%) Control 2 of 20 (10%)	Good engagement. They completed on average 5.3 modules (SD = 2.5) but were given on average of 7 (SD = 2.1) out of a possible 10 modules. TCS mean score was 33.8 (SD = 9.1) 2 weeks into treatment. CCSQ mean score was 23.8 (SD = 3.1), indicating good satisfaction.	Not reported	Not reported	All but one patient believed that the pregnancy-adaptation of the treatment was important and helpful.
Hayden et al. [61]	C/BT vs. supportive counseling	Not reported	Unclear	Unclear	Average therapy duration was 7.7 weeks (SD = 2.8), of a total of 10 weeks, and duration was not different between therapy groups.	Not reported	(−) Treatment outcome and participant demographics (age, race). (−) Attrition and demographics. (−) Attrition and baseline depression.	Not reported
Milgrom et al. [62]	C/BT vs. routine clinical care	Not reported	Intervention 11.11% Control unclear Reasons for nonuptake included one individual being hospitalized shortly after joining the study and suffered a reproductive loss at 25 weeks gestation, and one cited other health concerns as a reason.	Intervention 4 of 27 (14.81%) Control 6 of 27 (22.22%)	Of a possible eight sessions, women in the intervention attended an average of 6.30 sessions (SD = 2.91). The helpfulness of the intervention was rated 8.6/10 (SD = 1.4), and women's satisfaction with their treatment was rated 9/10 on average (SD = 0.9). All respondents to these questions (*n* = 19) reported that the intervention had been sufficient to address the problems they had been facing.	(+) There was a strong dose–response relationship between number of C/BT sessions and amelioration of depression.	Not reported	Not reported
Edge [39]	i-C/BT GSH vs. TAU	Methodology for measurement described, and their absence was also reported.	Intervention 0% Control 0%	Intervention 2 of 21 (9.52%) Control 1 of 22 (4.55%)	Good engagement with 86% (18 of 21 participants) completing all six sessions. Women visited the program on a mean of 20.5 occasions (SD = 10.6) and the mean number of sessions attended was 5.7 (SD = 0.7). Mean satisfaction ratings were in the moderately satisfied range (mean 3.1, SD = 0.60, range 2–4) on a 4-point scale. Similarly, mean ratings of the helpfulness of telephone coach calls were in the moderately helpful range (mean 3.2, SD = 0.89, range 1–4) on a 4-point scale.	Not reported	Not reported	Partners were signposted to resources, in recognition of their role, with 76% accessing this resource, however no improvement was found in women's relationships with their partners over the study period.
Milgrom et al. [64]	i-C/BT GSH vs. TAU	Methodology for measurement described, but no adverse events reported.	Intervention 3% Control unclear	Intervention 7 of 39 (17.95%) Control 3 of 39 (7.90%)	72% of participants (28 of 39) viewed all six sessions. Of the women who visited at least once, the mean number of sessions viewed was 5.6 (SD = 1.7). The helpfulness of the coach calls and treatment were rated on a scale from 0 to 3, with ratings of 2.56 (SD = 0.67), and 2.31 (SD = 0.89), respectively.	Not reported	(−) No association was found between attrition and the following baseline variables: depression; anxiety; perceived stress; marital functioning; CBT skills (Automatic Thoughts Questionnaire, Behavioral Activation for Depression Scale); maternal self-efficacy.	Participants and their partners were pointed towards a library of resources with content on PND and parenting. Participants rated the helpfulness of library articles and videos on a scale of 0 to 3, with a mean of 1.96 (SD = 1.04), and 1.87 (SD = 1.07), respectively. Partners were pointed to a partner support website and 39% (15 of 39 partners) accessed this, with participants rating the helpfulness of the partner website on a scale of 0 to 3, with a mean of 1.19 (SD = 1.02).
Ngai et al. [17]	Telephone-based C/BT vs. TAU	Not reported	Intervention 5.58% Control 0%	Intervention 10 of 197 (5.08%) Control 2 of 200 (1%)	86.8% completed all five sessions.	Not reported	Not reported	Parenting stress, quality of life, parenting self-efficacy and satisfaction were measured in secondary analysis. Improvements were made in these areas for the group receiving C/BT.
O'Mahen et al. [68]	C/BT vs. TAU	Not reported	Intervention 17% Control unclear	Intervention 8 of 30 (26.67%) Control 4 of 25 (16%). Reasons for dropout included not liking the session-by-session questionnaires, wanting more practical advice from the therapist, difficulties prioritizing self over family, and childcare difficulties.	Women received an average of 2.30 (SD = 2.16) sessions during pregnancy, and 5.35 (SD = 4.07) postpartum, of a total of 12 sessions. Women reported high levels of content applicability and treatment satisfaction in session-by-session assessments which used open-ended questions.	(+) Both treatment adherence and treatment satisfaction were associated with a reduction in depression scores.	(+) Applicability of the treatment material was associated with treatment satisfaction. (−) Applicability of the treatment material was not associated with depression at posttreatment, after controlling for depression at baseline. (+) Women more functionally impaired and women with higher baseline depression scores were less likely to engage with treatment. (−) Other participant demographic factors were not associated with treatment engagement (factors not clearly stated). (−) Participant psychological barriers to help seeking, such as avoidance (assessed via a 25-item measure with a 5-point Likert scale), were not associated with treatment engagement.	Barriers to adherence included struggling with care demands of a new baby, child illnesses and pregnancy-related pain, housing concerns, and lack of a private, safe home in which to meet.
O'Mahen et al. [69]	i-C/BT GSH vs. TAU	Not reported	Unclear	Intervention 281 of 462 (61.8%) Control 286 of 448 (63.84%)	Engagement was low, overall. There was good initial adherence reported in terms of session views, but this reduced significantly over time, with the greatest drop happening between sessions 1 and 2.	Not reported	(+) Women with a higher socioeconomic status were more likely to complete the baseline questionnaire. Qualitative information suggesting a link between hopelessness associated with depression and likelihood of adhering to treatment given that the hopelessness may permeate a belief around getting better.	There was a low response (22%) to a link to acceptability questions, but responses revealed acceptability and a role for internet-based interventions for women struggling with stigma and practical barriers to treatment. The qualitative study revealed that the anonymity of internet-based approaches was helpful in terms of stigma, but that stigma might still stop mums from engaging in homework practical activities. It also found that the perceived lack of social support as a mum is overwhelming, and a barrier to seeking help. The qualitative study (2015) highlighted themes, including the importance of guidance, and of personalized and relevant content, with examples that mums can relate to. The importance of accessibility was also raised, whilst noting that some would prefer a more structured routine particularly if they are lacking in motivation. Some noted a preference for face-to-face.
O'Mahen et al. [71]	i-C/BT GSH vs. TAU	Not reported	Intervention 2.44% Control unclear	Intervention 3 of 41 (7.32%) Control 8 of 42 (19.05%)	Women viewed a mean of 6.74 sessions (SD = 4.53), and completed 5.36 sessions (SD = 4.62), of a total of 12 sessions.	Not reported	(−) Telephone session adherence was not associated with the participant demographics of income, work status, relationship status, academic qualifications, number of children, or ethnicity. (+) Women with lower perceived support completed fewer program modules. (+) Women who were working or studying for a degree completed fewer program modules. (+) Women with poorer work and social adjustment baseline functioning and who were of a lower socioeconomic status opened fewer sessions.	The researchers note that women with complex life circumstances required more time to work through materials and supporters helped in terms of pacing the treatment content.
Pugh et al. [72]	i-C/BT GSH vs. waitlist	Not reported	Intervention 4% Control 0%	Intervention 4 of 25 (16.67%) Control 3 of 25 (12%)	Participants completed on average 5.92 of the seven modules (60% of the participants completed all seven modules). Over 80% of the participants reported that they liked the overall program. Participants also reported a high level of therapeutic alliance, giving a rating of 86.42% (TSQ)	Not reported	Not reported	Not reported
Rondung et al. [73]	i-C/BT GSH vs. TAU	Not reported	Intervention 19% Control unclear. Researchers note quite a few women declined participation because they did not accept randomization to either intervention, that is, they preferred standard care.	Intervention 43 of 127 (33.86%) Control 26 of 131 (19.85%)	Very low adherence in the guided i-C/BT group, with fewer than half of participants going on to the second module and less than one-third advancing to the third.	Not reported	(−) Participants defined as lost to follow-up were no different from the others who were not lost to follow-up, with regard to any preintervention characteristic or the level of fear of birth at screening.	The qualitative study [74]), reported view of i-C/BT being meaningful, helpful, flexible, private, but also not pinpointing fears, demanding, and resulting in feeling alone. The pregnancy itself brought an increased load affecting both family-life, work and the women's health which contributed to a low motivation to complete the i-C/BT, though some were motivated towards using i-C/BT. The range of views reported in the qualitative work suggest the importance of guidance and tailoring guidance.
Shaw et al. [76]	C/BT vs. Information/TAU	Reported as none	Intervention 0% Control 4.65%	Intervention 5 of 62 (8.06)% Control 2 of 43 (4.65%)	90% of mothers completed all six sessions and were satisfied with the treatment (satisfaction measure not noted).	Not reported	(+) Mothers with higher ratings of baseline NICU stress benefited more from the intervention compared with mothers who had lower ratings. (+) Mother's education and household income were found to be nonspecific predictors of the outcome (that is, they predicted outcome regardless of which intervention they received). Less educated mothers and mothers with lower household income showed a greater decline in trauma symptoms irrespective of whether they were in the intervention or comparison group. (−) The following variables were not found to be associated with treatment outcome: infant illness severity index, length of NICU stay, Traumatic Events Questionnaire, current major depressive episode, maternal age, white/non-white race, Hispanic ethnicity, and US born.	The researchers note that mothers in general reported greater satisfaction with the shorter than typical treatment (around 6 instead of 12 sessions), due to demands associated with having their infant in NICU.
Suchan, et al. [78]	i-C/BT GSH vs. TAU	Methodology for measurement described, and their absence was also reported.	Intervention 6.67% Control 3.03%	Intervention 4 of 30 (13.33%) Control 2 of 33 (6.06%)	75% (21/28) of the participants completed at least four of the five lessons. Pretreatment mean credibility factor score of the CEQ was 21.22 (SD = 3.38), and the mean score on the expectancy factor was 17.07 (SD = 3.77), with no differences between the treatment groups. Clients in the intervention treatment group demonstrated a significant increase in treatment credibility scores after treatment, mean of 23.58 (SD = 3.02). High levels of alliance with therapists according to the WAI.	Not reported	Not reported	Not reported
Trevillion et al. [79]	C/BT GSH plus TAU vs. TAU	Nine were reported but were not related to mental health or involvement in the trial.	Intervention 11.54% Control unclear	Intervention 2 of 26 (7.69%) Control 1 of 27 (3.70%). Reasons for withdrawal from treatment included inconvenience of the intervention alongside existing commitments, and symptoms resolved.	18 participants (69%) attended at least the minimum number of sessions (≥4 sessions). The overall median number of sessions attended was 6.5. Therapists demonstrated competence with regards to interpersonal effectiveness when fidelity was checked.	Not reported	Not reported	Not reported
Wozney et al. [80]	C/BT GSH vs. TAU	Not reported	Unclear	Intervention 5 of 32 (16.63%) Control 2 of 30 (6.67%)	The mean number of sessions across all intervention participants was 9.13 sessions (SD = 4.59), of a total of 12. A 10-item satisfaction questionnaire created by the research team was used and high satisfaction was found, including the helpfulness of coaches.	(+) A dose–response relationship between treatment engagement and outcome. Participants who engaged with a higher number of the intervention sessions were significantly more likely to no longer meet diagnostic criteria for depression. For every additional session of the intervention received, individuals had a 1.4 times greater chance of showing improvement.	(−) Other potential moderators, including cointervention, psychotropic medication, treatment duration, and booster sessions were not associated with treatment outcome.	Not reported

*Note:* (+) indicates an association and (–) indicates no association.

Abbreviations: BA, behavioral activation; C/BT, cognitive and/or behavioral therapy; CEQ, credibility/expectancy questionnaire; CSQ-8, clients satisfaction questionnaire-8; EPDS, Edinburgh postnatal depression scale; i-C/BT, internet-based CBT; MADRS-S, Montgomery and Asberg depression rating scale; NICU, neonatal intensive care unit; TAU, treatment as usual; TCS, treatment credibility scale; TSQ, treatment satisfaction questionnaire; WAI, working alliance inventory.

## Data Availability

The dataset is available from the corresponding author.

## Supporting Information

Supporting Information Search strategy example search.
