# Correction to “A Systematic Review of Factors Associated With Treatment Engagement and Outcome for Women in the Perinatal Period Receiving Individual Cognitive Behavioral Therapy (CBT) for Depression, Anxiety, and Trauma‐Related Disorders”

**DOI:** 10.1155/da/9761031

**Published:** 2026-02-03

N. A. Simon, J. Evans, D. Bispham, et al., “A Systematic Review of Factors Associated With Treatment Engagement and Outcome for Women in the Perinatal Period Receiving Individual Cognitive Behavioral Therapy (CBT) for Depression, Anxiety, and Trauma‐Related Disorders,” *Depression and Anxiety* 2025 (2025): 3698331, https://doi.org/10.1155/da/3698331.

In the article titled, “A Systematic Review of Factors Associated With Treatment Engagement and Outcome for Women in the Perinatal Period Receiving Individual Cognitive Behavioral Therapy (CBT) for Depression, Anxiety, and Trauma‐Related Disorders,” author Jonathan Jones was affiliated to Perinatal Community Mental Health Service, Cardiff and Vale UHB, Wales, Cardiff, UK, which is incorrect. The correct affiliation for this author is as follows:

University Library Service, Cardiff University, Cardiff, Wales.

The corrected list of affiliations is shown in the author information below:

Natalie A. Simon^1^, Jenna Evans^2^, Darcy Bispham^2^, Ffion Williams^2^, Jonathan Jones^3^, Neil P. Roberts^4^, and Cerith S. Waters^1,2^

^1^South Wales Clinical Psychology Training Program, School of Psychology, Cardiff University, Wales, Cardiff, UK

^2^Perinatal Community Mental Health Service, Cardiff and Vale UHB, Wales, Cardiff, UK

^3^University Library Service, Cardiff University, Cardiff, Wales

^4^Division of Psychological Medicine and Clinical Neuroscience, Cardiff University, Wales, Cardiff, UK

We apologize for this error.

