# Supplementary material for: A Systematic Review of Factors Associated With Treatment Engagement and Outcome for Women in the Perinatal Period Receiving Individual Cognitive Behavioral Therapy (CBT) for Depression, Anxiety, and Trauma-Related Disorders
Source: Depress Anxiety. 2025 Sep 18;2025:3698331. doi: 10.1155/da/3698331 (PMC12463511; doi:10.1155/da/3698331)
Supplement: Supporting Information — Search strategy example search. [file 3698331.f1.docx]

**Supplementary Material**

**S1 – Search strategy example search**

Example from Ovid via MEDLINE(R) ALL

Ovid via MEDLINE(R) ALL <1946 to November 15, 2022>

1 intrapartum period/ or antepartum period/ or perinatal period/ or postnatal period/ 49594

2 prenatal care/ 80822

3 maternal.mp. 883499

4 birth/ or birth trauma/ or pregnancy/ 1678968

5 intrapartum.mp. [mp=ti, ab, hw, tc, id, ot, tm, mf, tn, dm, dv, kf, fx, dq, bt, nm, ox, px, rx, ui, sy] 25596

6 intra partum.mp. [mp=ti, ab, hw, tc, id, ot, tm, mf, tn, dm, dv, kf, fx, dq, bt, nm, ox, px, rx, ui, sy] 1062

7 postpartum.mp. [mp=ti, ab, hw, tc, id, ot, tm, mf, tn, dm, dv, kf, fx, dq, bt, nm, ox, px, rx, ui, sy] 190731

8 post partum.mp. [mp=ti, ab, hw, tc, id, ot, tm, mf, tn, dm, dv, kf, fx, dq, bt, nm, ox, px, rx, ui, sy] 34962

9 prenatal.mp. [mp=ti, ab, hw, tc, id, ot, tm, mf, tn, dm, dv, kf, fx, dq, bt, nm, ox, px, rx, ui, sy] 492989

10 pre natal.mp. [mp=ti, ab, hw, tc, id, ot, tm, mf, tn, dm, dv, kf, fx, dq, bt, nm, ox, px, rx, ui, sy] 3940

11 antepartum.mp. [mp=ti, ab, hw, tc, id, ot, tm, mf, tn, dm, dv, kf, fx, dq, bt, nm, ox, px, rx, ui, sy] 17296

12 ante partum.mp. [mp=ti, ab, hw, tc, id, ot, tm, mf, tn, dm, dv, kf, fx, dq, bt, nm, ox, px, rx, ui, sy] 1066

13 perinatal.mp. [mp=ti, ab, hw, tc, id, ot, tm, mf, tn, dm, dv, kf, fx, dq, bt, nm, ox, px, rx, ui, sy] 260591

14 peri natal.mp. [mp=ti, ab, hw, tc, id, ot, tm, mf, tn, dm, dv, kf, fx, dq, bt, nm, ox, px, rx, ui, sy] 685

15 postnatal.mp. [mp=ti, ab, hw, tc, id, ot, tm, mf, tn, dm, dv, kf, fx, dq, bt, nm, ox, px, rx, ui, sy] 310293

16 post natal.mp. [mp=ti, ab, hw, tc, id, ot, tm, mf, tn, dm, dv, kf, fx, dq, bt, nm, ox, px, rx, ui, sy] 21801

17 pregnancy.mp. [mp=ti, ab, hw, tc, id, ot, tm, mf, tn, dm, dv, kf, fx, dq, bt, nm, ox, px, rx, ui, sy] 2098792

18 maternal.mp. [mp=ti, ab, hw, tc, id, ot, tm, mf, tn, dm, dv, kf, fx, dq, bt, nm, ox, px, rx, ui, sy] 883499

19 birth.mp. [mp=ti, ab, hw, tc, id, ot, tm, mf, tn, dm, dv, kf, fx, dq, bt, nm, ox, px, rx, ui, sy] 970340

20 childbirth.mp. [mp=ti, ab, hw, tc, id, ot, tm, mf, tn, dm, dv, kf, fx, dq, bt, nm, ox, px, rx, ui, sy] 75271

21 1 or 2 or 3 or 4 or 5 or 6 or 7 or 8 or 9 or 10 or 11 or 12 or 13 or 14 or 15 or 16 or 17 or 18 or 19 or 20 3354860

22 (anxiety or panic or phobic disorder or agoraphobia or obsessive compulsive disorder or obsessive behavio*).mp. [mp=ti, ab, hw, tc, id, ot, tm, mf, tn, dm, dv, kf, fx, dq, bt, nm, ox, px, rx, ui, sy] 1101432

23 (depress* or dysthymi* or melancholia or postnatal dysphoria or puerperal depression or major depression).mp. [mp=ti, ab, hw, tc, id, ot, tm, mf, tn, dm, dv, kf, fx, dq, bt, nm, ox, px, rx, ui, sy] 1967169

24 (Posttraumatic stress or post traumatic stress or PTSD or trauma related disorder* or complex trauma or DESNOS or acute stress disorder or stress reactions or trauma).mp. [mp=ti, ab, hw, tc, id, ot, tm, mf, tn, dm, dv, kf, fx, dq, bt, nm, ox, px, rx, ui, sy] 925924

25 exp anxiety/ or exp Anxiety Disorders/ or exp major depression/ or exp posttraumatic stress disorder/ or exp acute stress disorder/ 1080297

26 22 or 23 or 24 or 25 3400260

27 (therap* or Cognitive Therap* or CBT or Behavio* Therap* or Treatment or psychological therap*).mp. [mp=ti, ab, hw, tc, id, ot, tm, mf, tn, dm, dv, kf, fx, dq, bt, nm, ox, px, rx, ui, sy] 23833870

28 exp counseling/ 323799

29 27 or 28 23998840

30 (Randomized Control* Trial or randomised control* trial or Controlled Clinical Trial or Pragmatic Clinical Trial or Equivalence Trial or Clinical Trial, Phase III).mp. [mp=ti, ab, hw, tc, id, ot, tm, mf, tn, dm, dv, kf, fx, dq, bt, nm, ox, px, rx, ui, sy] 1967511

31 randomized controlled trials/ 398351

32 clinical trials/ 119355

33 Random Allocation.mp. 119825

34 RCT.mp. [mp=ti, ab, hw, tc, id, ot, tm, mf, tn, dm, dv, kf, fx, dq, bt, nm, ox, px, rx, ui, sy] 90459

35 30 or 31 or 32 or 33 or 34 2335848

36 21 and 26 and 29 and 35 6683

37 limit 36 to english language 6521

38 limit 37 to human 6213

39 limit 38 to humans 6213

40 limit 39 to "300 adulthood <age 18 yrs and older>" 6119

41 limit 40 to ("therapy (maximizes sensitivity)" or "therapy (maximizes specificity)" or "therapy (best balance of sensitivity and specificity)") 5952

42 remove duplicates from 41 4320

43 intrapartum period/ or antepartum period/ or perinatal period/ or postnatal period/ 49594

44 prenatal care/ 80822

45 maternal.mp. 883499

46 birth/ or birth trauma/ or pregnancy/ 1678968

47 intrapartum.mp. [mp=ti, ab, hw, tc, id, ot, tm, mf, tn, dm, dv, kf, fx, dq, bt, nm, ox, px, rx, ui, sy] 25596

48 intra partum.mp. [mp=ti, ab, hw, tc, id, ot, tm, mf, tn, dm, dv, kf, fx, dq, bt, nm, ox, px, rx, ui, sy] 1062

49 postpartum.mp. [mp=ti, ab, hw, tc, id, ot, tm, mf, tn, dm, dv, kf, fx, dq, bt, nm, ox, px, rx, ui, sy] 190731

50 post partum.mp. [mp=ti, ab, hw, tc, id, ot, tm, mf, tn, dm, dv, kf, fx, dq, bt, nm, ox, px, rx, ui, sy] 34962

51 prenatal.mp. [mp=ti, ab, hw, tc, id, ot, tm, mf, tn, dm, dv, kf, fx, dq, bt, nm, ox, px, rx, ui, sy] 492989

52 pre natal.mp. [mp=ti, ab, hw, tc, id, ot, tm, mf, tn, dm, dv, kf, fx, dq, bt, nm, ox, px, rx, ui, sy] 3940

53 antepartum.mp. [mp=ti, ab, hw, tc, id, ot, tm, mf, tn, dm, dv, kf, fx, dq, bt, nm, ox, px, rx, ui, sy] 17296

54 ante partum.mp. [mp=ti, ab, hw, tc, id, ot, tm, mf, tn, dm, dv, kf, fx, dq, bt, nm, ox, px, rx, ui, sy] 1066

55 perinatal.mp. [mp=ti, ab, hw, tc, id, ot, tm, mf, tn, dm, dv, kf, fx, dq, bt, nm, ox, px, rx, ui, sy] 260591

56 peri natal.mp. [mp=ti, ab, hw, tc, id, ot, tm, mf, tn, dm, dv, kf, fx, dq, bt, nm, ox, px, rx, ui, sy] 685

57 postnatal.mp. [mp=ti, ab, hw, tc, id, ot, tm, mf, tn, dm, dv, kf, fx, dq, bt, nm, ox, px, rx, ui, sy] 310293

58 post natal.mp. [mp=ti, ab, hw, tc, id, ot, tm, mf, tn, dm, dv, kf, fx, dq, bt, nm, ox, px, rx, ui, sy] 21801

59 pregnancy.mp. [mp=ti, ab, hw, tc, id, ot, tm, mf, tn, dm, dv, kf, fx, dq, bt, nm, ox, px, rx, ui, sy] 2098792

60 maternal.mp. [mp=ti, ab, hw, tc, id, ot, tm, mf, tn, dm, dv, kf, fx, dq, bt, nm, ox, px, rx, ui, sy] 883499

61 birth.mp. [mp=ti, ab, hw, tc, id, ot, tm, mf, tn, dm, dv, kf, fx, dq, bt, nm, ox, px, rx, ui, sy] 970340

62 childbirth.mp. [mp=ti, ab, hw, tc, id, ot, tm, mf, tn, dm, dv, kf, fx, dq, bt, nm, ox, px, rx, ui, sy] 75271

63 43 or 44 or 45 or 46 or 47 or 48 or 49 or 50 or 51 or 52 or 53 or 54 or 55 or 56 or 57 or 58 or 59 or 60 or 61 or 62 3354860

64 (anxiety or panic or phobic disorder or agoraphobia or obsessive compulsive disorder or obsessive behavio*).mp. [mp=ti, ab, hw, tc, id, ot, tm, mf, tn, dm, dv, kf, fx, dq, bt, nm, ox, px, rx, ui, sy] 1101432

65 (depress* or dysthymi* or melancholia or postnatal dysphoria or puerperal depression or major depression).mp. [mp=ti, ab, hw, tc, id, ot, tm, mf, tn, dm, dv, kf, fx, dq, bt, nm, ox, px, rx, ui, sy] 1967169

66 (Posttraumatic stress or post traumatic stress or PTSD or trauma related disorder* or complex trauma or DESNOS or acute stress disorder or stress reactions or trauma).mp. [mp=ti, ab, hw, tc, id, ot, tm, mf, tn, dm, dv, kf, fx, dq, bt, nm, ox, px, rx, ui, sy] 925924

67 exp anxiety/ or exp Anxiety Disorders/ or exp major depression/ or exp posttraumatic stress disorder/ or exp acute stress disorder/ 1080297

68 64 or 65 or 66 or 67 3400260

69 (therap* or Cognitive Therap* or CBT or Behavio* Therap* or Treatment or psychological therap*).mp. [mp=ti, ab, hw, tc, id, ot, tm, mf, tn, dm, dv, kf, fx, dq, bt, nm, ox, px, rx, ui, sy] 23833870

70 exp counseling/ 323799

71 69 or 70 23998840

72 (Randomized Control* Trial or randomised control* trial or Controlled Clinical Trial or Pragmatic Clinical Trial or Equivalence Trial or Clinical Trial, Phase III).mp. [mp=ti, ab, hw, tc, id, ot, tm, mf, tn, dm, dv, kf, fx, dq, bt, nm, ox, px, rx, ui, sy] 1967511

73 randomized controlled trials/ 398351

74 clinical trials/ 119355

75 Random Allocation.mp. 119825

76 RCT.mp. [mp=ti, ab, hw, tc, id, ot, tm, mf, tn, dm, dv, kf, fx, dq, bt, nm, ox, px, rx, ui, sy] 90459

77 72 or 73 or 74 or 75 or 76 2335848

78 63 and 68 and 71 and 77 6683

79 limit 78 to english language 6521

80 limit 79 to human 6213

81 limit 80 to humans 6213

82 limit 81 to "300 adulthood <age 18 yrs and older>" 6119

83 limit 82 to ("therapy (maximizes sensitivity)" or "therapy (maximizes specificity)" or "therapy (best balance of sensitivity and specificity)") 5952

84 remove duplicates from 83 4320
